# Anxiety, Depression, and Quality of Life Among Infertile Women: A Case-Control Study

**DOI:** 10.7759/cureus.55837

**Published:** 2024-03-09

**Authors:** Anupama Bahadur, Sukriti Kumari, Rajlaxmi Mundhra, Kavita Khoiwal, Anindya Das, Ayush Heda, Anjali Pathak, Sakshi Heda

**Affiliations:** 1 Obstetrics and Gynaecology, All India Institute of Medical Sciences, Rishikesh, Rishikesh, IND; 2 Psychiatry, All India Institute of Medical Sciences, Rishikesh, Rishikesh, IND

**Keywords:** stress, quality of life, mental health, infertility, depression, anxiety

## Abstract

Introduction

Pregnancy holds significant cultural and social value for women. However, women facing challenges in conceiving often grapple with emotional distress, including depression and anxiety. The connection between psychological elements (stress, anxiety, and depression) and infertility is complex, influenced by multiple factors, and bidirectional. Infertile women are more likely to develop mental illnesses, marital dissatisfaction, and impaired quality of life compared to the individuals of the fertile group. Thus, the study aimed to assess levels of anxiety, depression, and quality of life among infertile women compared to fertile women.

Methods

This case-control study conducted at a tertiary care center recruited 100 nulliparous women between the age group of 20 and 38 years with primary or secondary infertility, while those with male factor infertility were excluded. The control group (N=100) comprised normal parous women who had at least one child. The primary objective of the study was to assess the impact of infertility on the mental health and quality of life of women seeking infertility treatment. Outcome measures included standardized tools such as the WHOQOL-BREF questionnaire to assess the quality of life across multiple domains (e.g., physical, psychological, social, and environmental) as well as the Depression Anxiety and Stress Scale (DASS-21) to measure levels of anxiety, depression, and stress. Cronbach's alpha was used to measure the tool's reliability. A P value of <0.05 was considered statistically significant.

Results

Baseline sociodemographic parameters were comparable between the two groups. The mean age of infertile women was 30.6±3.9 years compared to 31.5±3.2 years in fertile women (P=0.076). Using the WHOQOL-BREF scale, we found that the quality of life was better in the fertile group compared to the infertile group through all the physical, psychological, social, and environmental domains (P<0.001). The infertile group had a significantly higher number of women with anxiety, depression, and stress. The questionnaires showed high internal reliability.

Conclusion

Infertile women experienced a lower quality of life in various domains, higher levels of anxiety, and increased rates of depression compared to fertile counterparts. The study findings underscore the multidimensional impact of infertility, emphasizing the need for comprehensive healthcare approaches to address the psychosocial challenges faced by women undergoing infertility treatment.

## Introduction

Pregnancy, and bringing a new life into the world, is regarded as a valued role for women all over the world [1]. As a result, women who are struggling to conceive frequently experience feelings of depression, anxiety, isolation, and loss of control [2]. In addition to the feeling of unfulfillment that these women often experience, they are also exposed to domestic violence and other forms of emotional trauma, especially in patriarchal societies [3].

Infertility is described as the inability to conceive after a year of consistent, unprotected sexual activity. The World Health Organization (WHO) identifies infertility as a major healthcare and social problem that leads to mental health issues like depression and anxiety, marital conflict, social isolation, and sexual dysfunction [4]. Compared to their male counterparts, women who are infertile appear to be more sensitive to stress. Couples who are struggling with infertility experience a heavy psychological load that negatively affects their quality of life [5].

Literature suggests that infertility and its association among females differs in diverse population groups, depending upon the demographic, economic, and social structure of the population under study [6]. Several studies have explored the quality of life in infertile women, predominantly employing descriptive approaches and relying on cross-sectional methodologies [7]. However, these studies have been limited by their lack of a comparison group, hindering the comprehensive analysis of the influence of infertility on various facets of life. This study was conducted to assess the quality of life and physical and mental health of women being treated for infertility.

## Materials and methods

Study overview

This case-control study was conducted from January 2018 to June 2019 at a tertiary care hospital in north India. Our aim was to study the anxiety, depression, and quality of life in infertile women compared to fertile women.

Ethical considerations

Ethical clearance was obtained from the Institute Ethics Committee (AIIMS/IEC/18/123).

Study criteria

After obtaining ethical clearance, women between 20 and 38 years of age willing to sign the informed written consent were recruited. Women with known psychiatric illness or diagnosed mental health issues or an episode of anxiety or stress in the past three months and women with current use of alcohol or drugs were excluded. Cases comprised 100 nulliparous women attending the outpatient department of the hospital seeking treatment for primary or secondary infertility, while couples with male factor infertility were excluded. Controls were 100 normal parous women who had at least one child older than one year. Pregnant women and women less than four months from the last childbirth were excluded as controls. Infertility was defined as not being able to achieve pregnancy after one year of having regular, unprotected intercourse.

Procedure

A detailed history was taken followed by clinical examination, including relevant anthropometric measurements, gynecological examination, systemic examination, and examination for secondary sexual characteristics. The participants were asked to complete a questionnaire that comprised of tools for assessing the level of anxiety, depression, and the overall quality of life. All the questions were translated into Hindi to avoid any misunderstanding because of the language barrier and explained properly to each participant.

Assessments

We used various tools to assess these parameters and the quality of life of infertile women, namely, the WHO Quality of Life (WHOQOL-BREF) and Depression Anxiety and Stress Scale (DASS-21). WHOQOL-BREF was used to assess the quality of life. It comprises 26 questions divided into four domains: physical, psychological, social, and environmental. The scores were transformed on a 0-100 scale [8]. Permission to use the WHOQOL instruments was obtained. DASS-21 is a set of 21 questions with seven items per scale and was used to measure the negative emotional states of anxiety, stress, and depression [9].

Sample size

Convenience sampling (time-bound study) was used to obtain the sample size.

Statistical analysis

Continuous normally distributed variables were compared using independent t-tests, while continuously distributed nonparametric variables were compared using the Mann-Whitney U test. Comparing categorical variables was done using the chi-square test. Cronbach's alpha was used to measure the tool's reliability. A P of <0.05 was considered statistically significant.

## Results

A total of 200 women were recruited with 100 infertile women and 100 normal controls. The women in both groups were comparable in terms of education, occupation, family structure, socioeconomic status, and other baseline characteristics, as shown in Table 1.

**Table 1:** Baseline characteristics of the study population

Baseline characteristics	Infertile group (N=100), n (%)	Fertile group (N=100), n (%)
Age (years) (mean ± SD)	30.6 ± 3.9	31.5 ± 3.2
Education status		
Primary education	65 (65)	54 (54)
Secondary education	26 (26)	46 (46)
Higher education	9 (9)	0 (0)
Socioeconomic status		
Upper middle	20 (20)	27 (27)
Lower middle	30 (30)	30 (30)
Upper lower	32 (32)	24 (24)
Lower	18 (18)	19 (19)
Menstrual cycles		
Regular	83 (83)	69 (69)
Irregular	17 (17)	31 (31)
Abortion history		
No abortions	74 (74)	79 (79)
One abortion	22 (22)	11 (11)
Two abortions	3 (3)	7 (7)
Three abortions	1 (1)	3 (3)
Contraception		
Yes	2 (2)	45 (45)
No	98 (98)	55 (55)

Mean age was comparable between cases and controls (30.6 ± 3.9 versus 31.5 ± 3.2 years; P=0.076). Menstrual irregularity was higher in fertile women compared to infertile women (P=0.015). Most patients in the present study had primary infertility (74%), while the rest were nulliparous females with secondary infertility.

The factors affecting fertility among infertile women were also identified. Tubal factors were the most common (45%), followed by polycystic ovary syndrome (PCOS) (18%), grade IV endometriosis (7%), uterine fibroids (4%), and endocrine factors (4%), while 22% cases had unexplained infertility.

The quality of life and status of anxiety and depression were assessed using questionnaires, and fertile women fared better in terms of quality of life and mental status compared to the infertile women, as shown in Table 2. Infertile women scored lower in all the domains of WHOQOL-BREF, including physical, psychological, social, and environmental. The assessment tools demonstrated high internal reliability, enhancing confidence in the observed differences between infertile and fertile women. Based on the DASS-21 scale, approximately 29% of the infertile women were found to be stressed, and there was a high prevalence of anxiety (63%) and depression (67%). The psychological parameters assessed (e.g., stress, anxiety, and depression) were found to be significantly higher in infertile women compared to the fertile group.

**Table 2:** Comparison of quality of life and mental health status between the groups DASS-21: Depression Anxiety and Stress Scale. Transformed World Health Organization Quality of Life Brief Version (WHOQOL-BREF) domain scores are shown ranging from 0 to 100 and are scaled in a positive direction (a higher score denotes a higher quality of life).

Scales	Reliability (Cronbach’s alpha)	Infertile women (N=100)	Fertile women (N=100)	P value
WHOQOL-BREF (mean ± SD)	0.95			
Physical domain		56.5 ± 6.4	77.7 ± 7.2	<0.001
Psychological domain		54.2 ± 6.5	80.4 ± 6.1	<0.001
Social domain		57.2 ± 10.1	74.7 ± 10.3	<0.001
Environment domain		57.7 ± 7.6	76.3 ± 6.4	<0.001
DASS-21 stress scale	0.76			0.001
Stress		29 (29)	4 (4)	
Normal		71 (71)	96 (96)	
DASS-21 anxiety level	0.65			<0.01
Anxiety		63 (63)	20 (20)	
Normal		37 (37)	80 (80)	
DASS-21 depression level	0.50			0.01
Depression		67 (67)	2 (2)	
Normal		33 (33)	98 (98)	

## Discussion

The WHO estimates that 60-80 million couples in the world are infertile. It is not only a gynecological problem but also a bio-psychosocial health problem that causes social distress, marital conflict, sexual dissatisfaction, and psychological issues like depression, anxiety, and social isolation [10]. There are higher levels of anxiety and depression among women compared to men during infertility treatment [4]. Infertility is a multidimensional problem, which lowers the quality of life and affects the psychological, environmental, physical, and social functioning of an individual [11]. It has detrimental psychosocial and cultural repercussions and causes, anxiety, social exclusion, deprivation, instability in marriages, loss of self-esteem, loss of gender identity, loss of control, and feelings of shame and self-blame [12]. The present study evaluated the impact of infertility on the mental health of women at a tertiary health care center in Uttarakhand, India, and showed that infertile women have a diminished quality of life across multiple dimensions. Several aspects of quality of life, including physical, mental, social, and environmental well-being, were found to be lower among infertile women compared to their fertile counterparts. This study aligns with previous research demonstrating this cause-and-effect relationship [13].

The mean age of infertile women in our study was 30.6 years and that of fertile women was 31.52 years, and both groups were comparable. This was in agreement with the observations made by Dyer et al. and Bakhtiyar et al. in their studies, where there was no significant difference in the mean age of fertile and infertile women presenting in the outpatient department (OPD) [7,13]. In contrast, Aduloju et al. observed a significantly higher mean age of fertile women compared to that of infertile women in their study [14]. This difference in observation could be because infertile women presented relatively earlier to their OPD than fertile women.

Based on the WHOQOL-BREF, we observed that infertility affects the physical and psychological health of women, disturbs their social relationships and environment, and, consequently, lowers the quality of life of these women. Bakhtiyar et al. observed that while infertile women experience a relatively low quality of life in physical, mental, and environmental health subscales than fertile women, they attained higher scores in the social domain. The reason behind this could be that infertile women in Iran receive increased social support, stemming from various factors such as personal connections or familial ties [7]. Aduloju et al. reported worse quality of life, in the mean scores of physical, psychological, and environmental functions in the infertile group [14].

We found that based on the DASS-21 scale, infertile women were more depressed compared to the fertile controls, with the difference being statistically significant. This was consistent with the results of studies by Lakatos et al. and Lund et al. [15,16]. Lakatos et al. found that 44.8% of moderate to severe depression in cases with infertility, while a high level of anxiety was reported in around 40% of cases. Hasan et al. evaluated the mental health status of Bangladeshi women undergoing fertility treatment and reported a high prevalence of depression, anxiety, and stress (59.7%, 55.0%, and 48.7%, respectively) [17]. Shahraki et al. found that women with primary infertility suffered more from depression than those with secondary infertility [18]. Social factors have the potential to impact infertility, and it is reasonable to anticipate that the occurrence of mental health disorders among individuals facing infertility may differ across various cultures. Various studies globally have explored the relationship between infertility and anxiety. For example, anxiety symptoms were observed in 52-83.8% of infertile women in China, 33% in Hong Kong, 86.6% in Iran, 67% in Spain, and 24.9% in the Netherlands, Belgium, and France [1].

Xiaoli et al. in their cross-sectional study evaluating the quality of life in infertile women found that, when compared to fertile women, infertile women had significantly lower overall and comprehensive quality of life ratings and greater anxiety levels [19]. This was consistent with the present study as we found higher stress and anxiety in the infertile group.

The strength of our study lies in the fact that two different tools with good reliability were used to assess the levels of stress, depression, anxiety, and quality of life. One of the limitations of the study was that it was a hospital-based study and may limit the generalizability of the findings to the wider community. It is anticipated that women visiting the clinic may receive social support from their spouses. Cultural beliefs regarding the importance of childbearing within marital relationships may vary, potentially impacting quality of life. However, this aspect was not explored in the present study. A community-based approach might uncover distinct findings regarding the quality of life among infertile women who choose alternative treatment options or opt for no treatment at all. Hence, future studies that adopt a community-based and multicultural approach may provide deeper insights into the influence of cultural beliefs on the quality of life among infertile women.

## Conclusions

The present study sheds light on the significant psychological burden experienced by women struggling with infertility. The findings reveal that infertile women face a lower quality of life across various domains, including physical, psychological, social, and environmental well-being, compared to their fertile counterparts. The prevalence of anxiety and depression among infertile women underscores the need for comprehensive psychosocial support and healthcare interventions in infertility management. The study findings underscore the multidimensional impact of infertility, emphasizing the need for comprehensive healthcare approaches to address the psychosocial challenges faced by women undergoing infertility treatment. Future research should adopt a community-based and multicultural approach to further explore the influence of cultural beliefs on the quality of life among infertile women and explore interventions and support mechanisms to mitigate the psychological burden experienced by infertile women.
